# Variation in Lithic Technological Strategies among the Neanderthals of Gibraltar

**DOI:** 10.1371/journal.pone.0065185

**Published:** 2013-06-06

**Authors:** Ceri Shipton, Christopher Clarkson, Marco Antonio Bernal, Nicole Boivin, Clive Finlayson, Geraldine Finlayson, Darren Fa, Francisco Giles Pacheco, Michael Petraglia

**Affiliations:** 1 School of Social Science, University of Queensland, Brisbane, Queensland, Australia; 2 The Gibraltar Caves Project, Gibraltar Museum, Gibraltar; 3 School of Archaeology, University of Oxford, Oxford, England, United Kingdom; 4 Department of Social Sciences, University of Toronto at Scarborough, Toronto, Ontario, Canada; Universidad Autonoma de Barcelona and University of York, Spain

## Abstract

The evidence for Neanderthal lithic technology is reviewed and summarized for four caves on The Rock of Gibraltar: Vanguard, Beefsteak, Ibex and Gorham’s. Some of the observed patterns in technology are statistically tested including raw material selection, platform preparation, and the use of formal and expedient technological schemas. The main parameters of technological variation are examined through detailed analysis of the Gibraltar cores and comparison with samples from the classic Mousterian sites of Le Moustier and Tabun C. The Gibraltar Mousterian, including the youngest assemblage from Layer IV of Gorham’s Cave, spans the typical Middle Palaeolithic range of variation from radial Levallois to unidirectional and multi-platform flaking schemas, with characteristic emphasis on the former. A diachronic pattern of change in the Gorham’s Cave sequence is documented, with the younger assemblages utilising more localized raw material and less formal flaking procedures. We attribute this change to a reduction in residential mobility as the climate deteriorated during Marine Isotope Stage 3 and the Neanderthal population contracted into a refugium.

## Introduction

When chipping stone to create sharp edged tools, there are a wide range of strategies that a knapper may employ. The factors influencing the choice of knapping strategy include downstream effects from the selection of particular types of stone and clast morphologies, as well as the cultural repertoire, foraging methods and mobility of the hominin group. Understanding knapping strategies can therefore inform us about several aspects of hominin behaviour. In this study we look at knapping strategies among a particularly iconic set of hominins: the Neanderthals of Gibraltar, who are both one of the most comprehensively studied and latest surviving of all Neanderthal populations.

**Figure 1:**
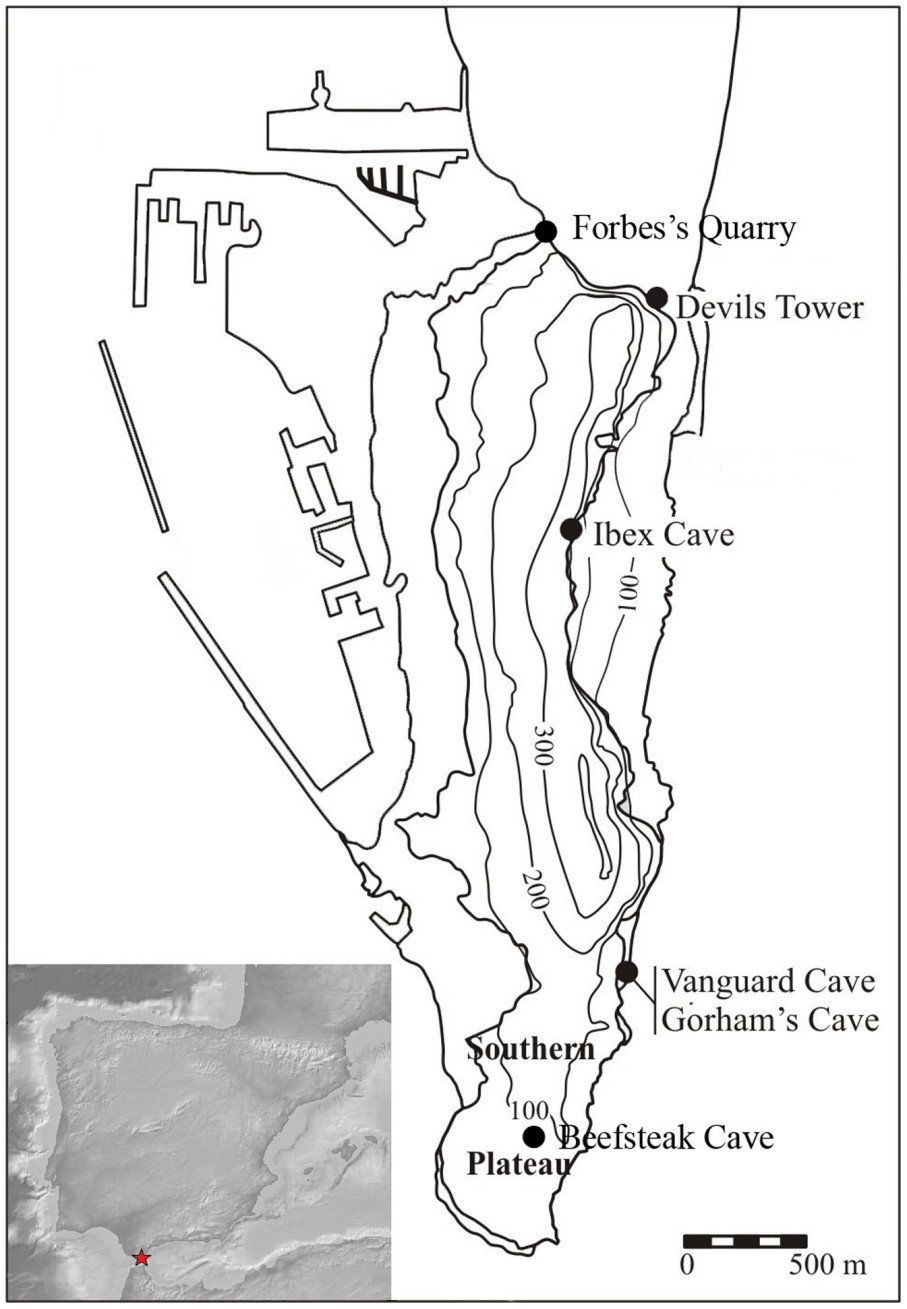
The location of sites mentioned in the text. Inset: the location of Gibraltar on the Iberian Peninsula.

The Rock of Gibraltar is a limestone klippe peninsula at the southern tip of Iberia (Figure 1) and represents the south-western extremity of the Neanderthal range. Both wave and solutional erosion have created a series of caves in the klippe, particularly on its more exposed eastern side, which were inhabited by Neanderthals and then *Homo sapiens* over the last 100 thousand years. Gibraltar is home to some of the world’s most significant Neanderthal sites. The region is historically significant as one of the first discoveries of Neanderthal skeletal remains was made in Forbes Quarry in 1848 [1], [2]. Important dietary information has been obtained from the Gibraltar caves, including the exploitation of a range of terrestrial and marine species unparalleled at other Neanderthals sites [3], [4]. Gibraltar also boasts having the youngest Mousterian sites in Europe, suggesting that the area served as a refugium for the final Neanderthals [5], [6].

The association between Mousterian technology and the Neanderthals is well documented across Europe and Gibraltar itself has played a role in establishing the link [5], [7]. The excavation of Devil’s Tower, a rockshelter on the north end of Gibraltar (Figure 1), produced a Neanderthal cranium in association with a Mousterian industry [8]. Although no Neanderthal remains were recovered from the caves described in this paper (which are just 3 km from Devil’s Tower), we assume that the Mousterian artefacts were made and used by Neanderthals.

The Gibraltar caves have been subject to excavations by a number of teams. Previous studies of the lithic assemblages have documented artefact typologies, reduction sequences, spatial patterns and putative functions [8], [9], [10], [11], [12], [13], [14], [15]. Here, we synthesise patterns in Mousterian lithic technology from Gibraltar, using published information and our own data. As a well documented refugium, Gibraltar presents an ideal opportunity to examine how lithic technology is adapted in response to changes in climate and hominin range size. In general more formal technologies tend to be used by more mobile hunter-gatherer groups, while hunter-gatherers with smaller ranges tend to invest less in technological adaptations [16], [17]. Core reduction strategies, flake platform preparation, ratios of different artefact classes and raw material selection have been particularly informative elsewhere in studies of Neanderthal mobility and technological adaptations e.g. [18], [19], [20], [21]. To test for technological variation on Gibraltar we recorded raw materials, artefact classes, flake platform types, and a suite of metric measurements to characterize the technology of lithic cores.

Various materials suitable for lithic manufacture are available on Gibraltar, the lowest quality of which is the limestone of The Rock itself. A quartzite outcrop occurs on the western side of The Rock, with primary sources of quartzitic sandstone available within 10km of Gibraltar [22]. Although these materials were utilised, the rounded cortex on most of the artefacts indicates they were procured as water-worn cobbles, which are readily available on the beaches [9], as well as from now submerged rivers and alluvial fans [22]. Occasional instances of more angular cortex may have been procured from now submerged pillars of quartzite 20 m below current sea level in front of Governor’s Beach on the eastern side of The Rock [22]. Previous studies have described quartzite and quartzitic sandstones [22], [15]. Though we suspect that many of these materials are in fact silcrete, we group these raw materials under the label quartzite for consistency with previous publications. Various colours of chert may be found as beach pebbles, embedded within several fossil beaches higher up The Rock [23] from sources that are currently submerged, and as primary veins in The Rock [22]. At the northern end of Gibraltar between Devil’s Tower and Forbes Quarry there is a source of heavily fractured dark grey chert [9], [22]. Red chert (sometimes called jasper) and green chert are available from the Devil’s Bellows, also towards the north of the Peninsula [11]. These cherts occur as pebbles and cobbles on the beaches, and the presence of rounded cortex on some artefacts indicates that they were exploited as such. Chemical composition indicates that while some of the chert derives from these marine sources, some was also obtained from inland Iberia [24]. Angular cortex on a honey coloured chert suggests procurement from a more primary source which is not known on Gibraltar. The nearest known source of this material is in terrace deposits 17km to the north-west of Gibraltar [15]. We may thus distinguish between four classes of raw material on Gibraltar: local limestone, local quartzite, local chert and introduced chert.

In this article we examine artefacts from four different caves: Vanguard, Beefsteak, Ibex and Gorham’s (Figure 1). Each cave provides its own signature of Neanderthal behaviour, enabling an assessment of spatial variation in the occupation of Gibraltar. Gorham’s Cave has a long occupation sequence, allowing us to look at diachronic change, in particular whether the late Marine Isotope Stage (MIS) 3 artefacts reflect continuity with the preceding sequence or an intrusive tradition.

### Vanguard Cave

Vanguard Cave is one of a series of caves on Governor’s Beach, which is on the south-east side of Gibraltar. Optically Stimulated Luminescence (OSL) dating indicates Middle Palaeolithic occupation mainly took place during MIS 5, after which time the cave became filled with sand [25], although radiocarbon dates suggest limited occupation may have extended into MIS 4 and 3 [26].

In the Middle Area of Vanguard cave three occupation horizons have been identified [27], each of which is associated with lithic artefacts [13]. OSL samples from contexts immediately overlying these occupation layers yielded dates of 118 and 121.6 kya [25]. All three horizons contain artefacts of quartzite, chert (including red chert) and limestone (Table 1). The lowest occupation horizon contains just 37 artefacts with the presence of two flakes with centripetal dorsal scar patterns and one facetted platform suggesting some use of discoidal and/or Levallois technology. The low density of artefacts in all three horizons suggests sporadic occupation.

**Table 1:** Breakdown of artefacts by raw material in Vanguard Cave Middle Area.

	Quartzite	Chert	Limestone	Other	Total
**Upper Horizon**	60 (59%)	27 (27%)	13 (13%)	1 (1%)	101
**Intermediate** **Horizon**	79 (44%)	76 (42%)	20 (11%)	6 (3%)	181
**Lower Horizon**	30 (81%)	4 (11%)	2 (5%)	1 (3%)	37

Data from Barton [13].

The intermediate occupation horizon contains two hammers of quartzite and one of sandstone, which, along with 46% of artefacts being smaller than 15 mm and the refitting of some chert flakes, suggests some *in situ* knapping [13]. The two cores recovered are both multiplatform, including one of chert and one of limestone. The limestone core has 14 scars on it and the 18 flakes and flaked pieces of limestone from this horizon may have been struck off this core. The core has a mean platform angle of 100° (taken on the last surface to be flaked), it exhibits no platform preparation and it is one of the largest cores found anywhere on Gibraltar, weighing 529 g. The lack of shaping and platform preparation on the core indicates that it was flaked opportunistically with little consideration for prolonging its use-life through the maintenance of low platform angles; hence it was discarded while still large. A core rejuvenation flake on red chert indicates this expedient flaking strategy was not applied to chert, instead effort was made to increase the use-life of chert clasts. Interestingly there is a sole flake of honey-coloured chert with a centripetal dorsal scar pattern, which at 54 mm long is larger than any of the other chert flakes [13]. It was likely transported to the site in its present form rather than being produced there. There is a hearth in this horizon and remains of seal, ibex and red deer, that show evidence of butchery with stone tools [27].

In the upper occupation horizon the presence of two quartzite cobble hammerstones, one of which refits from two halves, suggests some knapping took place here [13]. Just over half the artefacts were quartzite, with many of the quartzite pieces less than 15 mm in length. The only cores from this area were of limestone, one of which was discoidal and the other multiplatform. There are only a few plain limestone flakes (N = 5) and some flaked pieces (N = 6) which accords with the low number of flake scars (N = 5) on the multiplatform core. However, the discoidal core has 22 scars, suggesting smaller limestone flakes may not have been differentiated from the unmodified limestone of the cave during excavation. Chert artefacts are also present including the red and greyish-green varieties available on Gibraltar. The only flakes with complex centripetal dorsal scar patterns are in chert and the absence of any chert cores suggests these artefacts were part of a longer, more spatially distributed reduction sequence than either the quartzite or the limestone.

The Northern Alcove in Vanguard Cave, which is approximately the same level as the three occupation horizons also contained artefacts of quartzite, chert (including red chert), and limestone, and is associated with a hearth [10].

A hearth located in the upper part of Vanguard Cave, dated to 108.5 kya [25], is associated with shellfish remains and lithics. The lithics may be divided into two groups, a dense concentration of quartzite artefacts and 5 chert pieces [10] [13]. The chert artefacts are comprised of 3 retouched pieces and a plain flake on dark grey chert, and an *éclat debordant* on dark red chert, and were likely introduced as finished artefacts. It is suggested that some of these chert pieces were used as shucks for opening the associated shellfish [10]. The quartzite artefacts are numerous (N = 1084), largely concentrated in a dense c. 1 m^2^ area, and they include refits and 997 artefacts <15 mm in maximum dimension. All these factors indicate that they represent a discrete knapping episode, with the low frequency of thermal modification showing that this took place after the associated fire had died down [13]. Quartzite cobbles are available from the beach in front of Vanguard Cave and this artefact scatter was probably generated from such a source [13]. There are three cores in the scatter, two of which are multiplatform and the third a discoidal core. The discoidal core may have passed through a Levallois stage, as indicated by the presence of a Levallois flake and a centripetal flake with a facetted platform.

### Beefsteak Cave

Beefsteak Cave is located near to Europa Point at the southern tip of Gibraltar. Uranium series dating of layer D, which overlies Middle Palaeolithic artefacts in layers C and B, produced a date of 98.8±15.5 kya [14]. The artefact counts are extremely low for both layers C and B (Table 2) with most artefacts being retouched. This suggests that occupation of Beefsteak Cave was ephemeral and that artefacts were brought to the site rather than produced there. The two cores from layer B are morphologically and technologically very similar to each other. Both are made on small (35.98 and 37.29 mm in length) ovoid pebbles of chert with rounded cortex, suggesting that the chert was procured from the surrounding local beaches. Both cores have two interdependent hierarchical surfaces, with the lower surface having been faceted to provide a strong platform for bidirectional flaking of the upper surface, along the long axis of the pebble. The similarity of these two cores corroborates the suggestion that the occupation at Beefsteak represents discrete and brief episodes.

**Table 2:** Lithic artefacts from Beefsteak Cave.

Layer	Raw Material	Flakes	Retouched Flakes	Fragments	Cores
**C**	Chert		4		
	Quartzite		2		
**B**	Chert		4	6	2
	Quartzite	1		3	

Data from Giles et al., [14].

### Ibex Cave

**Figure 2:**
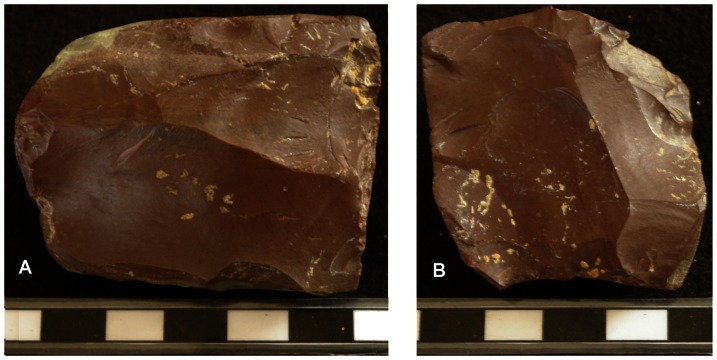
Two Levallois cores in red chert from Ibex Cave. A: recurrent bidirectional Levallois IBE 046. B: recurrent centripetal Levallois, IBE 053.

Ibex cave is located high on the eastern side of Gibraltar about halfway along the length of The Rock. Tooth enamel from a layer underlying Mousterian artefacts was dated using Electron Spin Resonance to 37 kya (early uptake (EU)) or 49 kya (linear uptake (LU)) [28]. Out of a total of 96 lithics from Ibex cave, 89 are of chert and the majority of these are dark red in colour. The remaining artefacts are six limestone flakes (that could have resulted from roof fall), and a hammerstone [11]. Three cores of the red chert were found in the cave, all of which may be classified as recurrent Levallois (Figure 2). Two of the cores were flaked centripetally and have refitting preferential flakes, while the third core was flaked bidirectionally. The cores are all between 4 and 6 cm in length and all exhibit platform faceting and overhang removal. There are no retouched flakes from Ibex Cave, while the presence of many cortical flakes (22%), suggests the complete reduction sequence of the red chert pebbles may have taken place here. The low diversity in raw material, the presence of refits, the high proportion of cortical flakes, the presence of 13 flaked pieces less than 5 mm long, and the similarity in the technology of the cores all suggest the lithic artefacts at Ibex represent a single occupation episode.

### Gorham’s Cave

Adjacent to Vanguard Cave on Governor’s Beach is the larger Gorham’s Cave. Micromorphology indicates that he cave was occupied intermittently by both hominins and hyenas [29]. The earliest excavations at Gorham’s were made by Waechter in the middle part of the cave in the 1950s. Three radiocarbon dates for the uppermost Mousterian level in this excavation produced dates in the region of 47–49 kya (all radiocarbon dates presented in this article are calibrated) [30], [26]. ESR dating on teeth from the Mousterian layers gave LU ages ranging from 26–62 kya for the Mousterian sequence [31]. The middle area of the cave was re-excavated by the Gibraltar Caves Project in the 1990s, with radiocarbon ages for the Mousterian occupation spanning 40 to 50 kya [26]. ESR dates for the Mousterian also gave this range, with ages from 39.4 to 50.8 kya [32]. The most recent radiocarbon program gave stratigraphically ordered dates from 48–33 kya for the upper part of the Mousterian occupation in the middle area [33]. New single grain OSL ages spanning most of the Mousterian sequence have produced stratigraphically ordered ages from 38.5 to 67.9 kya [34]. Since 1999 a new excavation campaign led by the Gibraltar Museum has been carried out in the upper area at the back of Gorham’s Cave. This excavation has revealed the youngest Mousterian occupation yet known anywhere in Europe, with a time span of 35 to 28 kya [5], [6] contra [35]. After 28 kya there is a hiatus in human activity before the cave is reoccupied by makers of the Solutrean facies of the Upper Palaeolithic [6]. The spatial clustering of artefacts of similar raw material throughout the Gorham’s Cave sequence and the presence of refits indicates the high integrity of the deposits and that knapping took place in the cave [15].

Based on stratigraphy, the Gorham’s Cave Mousterian sequence may be divided into six main phases. The lowermost phase contains few artefacts and is undated so will not be discussed further. The next phase comprises the upper Sands and Stony Lenses member (SSLm) which is divided into six subunits and may be correlated with Waechter’s layers L, M, O and P [36]. Two subunits for this member have OSL ages of 56.5 and 67.9 kya [34]. The use of the Levallois technique is clearly apparent in this member with some Levallois points recovered [15], as well as several Levallois cores. The Levallois cores include preferential centripetal forms as well as recurrent Levallois flaked centripetally, bidirectionally and unidirectionally. Flakes with facetted butts are numerous, where systematically recorded they comprise 25% of flakes and, correspondingly, flakes with prepared platforms constitute 21% of the assemblage from Waechter’s layers L, M, O and P (Table 3). Hammerstones and the presence of small chips <15 mm in length indicate on site knapping in some subunits. Retouched artefacts comprise 1.1% of the combined Waechter and more recent assemblages (Table 3), with types including notches, scrapers and burins. Chert dominates the raw materials (Table 4) including the Levallois cores, although quartzite was also used for this technique, while a single limestone core was flaked unifacially. The non-local honey coloured chert appears frequently in this member (Table 4), including four large flakes (>50 mm) with facetted platforms, a core rejuvenation flake from subunit 5 [15], and a blade 126 mm long from subunit 6.

**Table 3:** Proportions of platform preparation and retouch on flakes from Gorham’s Cave.

Layer	Percent Facetted Butts (Total N)	Equivalent Waechter Layers	Prepared Platforms (Total N)	Combined Retouched Percent
**SSLm**	25% (56)	L, M, O, P	21% (1997)	1.1% (2232)
**LBSm**	12% (61)	K	14% (1351)	2.9% (1774)
**BeSm**	12% (121)	H	N/A	3.5% (259)
**UBSm**	7% (41)	G	4% (4965)	1.2% (5120)
**Layer IV**	9% (30)	N/A	N/A	6.7% (165)

Data from Waechter [9], Barton & Jennings [15], Colcutt & Currant [36] and Pacheco et al. [14].

**Table 4:** Breakdown of raw materials in the Mousterian layers of Gorham’s Cave.

	Non-Local Chert	Chert	Quartzite	Limestone	Total
**Layer IV**	0	59 (33%)	118 (66%)	1 (1%)	178 (100%)
**UBSm**	5 (5%)	38 (38%)	56 (56%)	1 (1%)	100 (100%)
**BeSm**	9 (4%)	84 (36%)	135 (59%)	2 (1%)	230 (100%)
**LBSm**	22 (7%)	165 (55%)	104 (35%)	8 (3%)	299 (100%)
**SSLm**	19 (11%)	113 (64%)	31 (18%)	13 (7%)	176 (100%)

Quartzite includes quartzitic sandstone and silcrete. Data from Barton & Jennings [15] and Pacheco et al. [14].

Overlying the Sands and Stony Lenses member is the Lower Bioturbated Sands member (LBSm), which has numerous coarse and fine facies. Five radiocarbon dates place the age of this member at c. 47.5 kya [33]. Levallois technology is present in this member with recurrent bidirectional core forms, core rejuvenation flakes and Levallois flakes and points. A range of less formal core types are also present including facetted unidirectional and centripetal discs (distinguished from Levallois by a lack of shaping of the main flaking surface), classic discoidal cores, and occasional multi-platform and single platform cores. Both chert and quartzite cores are well represented. A moderate proportion of flakes have prepared platforms (Table 3) and again hammers and microdebitage indicate on site knapping. An artificially smoothed elongate cobble from this member has been interpreted as an abrader, while two ungulate long bone fragments are described as retouchers [15]. Retouched artefacts comprise 2.9% of the combined flake assemblages (Table 3) and include scrapers, notches, burins, Mousterian points and a denticulate. The Lower Bioturbated Sands have a similar raw material distribution to the member below with chert dominating over quartzite, however there is more use of quartzite here and less use of the non-local chert. One particular subunit has 12 flakes of honey coloured chert, five of which have centripetal dorsal scar patterns, a further three are Levallois flakes, and two are retouched artefacts [15].

The next member going up the sequence is the Bedded Sands (BeSm), which date to around 46 kya [33]. Levallois products are present in this member, along with a moderate proportion of facetted flakes (Table 3), but as yet no Levallois cores have been found. Most of the cores are discoidal with facetted discs also present from which the Levallois products could have been derived. Refits, small chips and longitudinally broken flakes demonstrate *in situ* knapping, with 39% of flakes having cortex [15] indicating the early stages of reduction were carried out here. Retouched artefacts comprise 3.5% of the flake assemblage (Table 3) and include denticulates and amorphous pieces. The use of quartzite over chert again increases in this member over the previous one, with non-local chert becoming rarer (Table 4). This level is equivalent to Waechter’s layer H, although he regards the artefacts from this level as being intrusive from the rich overlying layer G.

The most recent Mousterian member in the middle area of Gorham’s Cave is the Upper Bioturbated Sands member (UBSm). The three lower subunits of this member have Mousterian artefacts with radiocarbon dates for these subunits ranging from 45–34 kya [33]. This member is equivalent to Waechter’s layer G [36]. Levallois products are evident at low frequencies in this level, with two Levallois points and one Levallois flake recorded in the Stringer and Barton excavations [15]. However, very few Levallois cores were recovered, with Waechter producing just nine preferential Levallois specimens out of 150 cores, and no Levallois cores found in the more recent excavations [9], [15]. Instead, discoidal and multiplatform cores dominate both assemblages. Correspondingly, very few flakes from this layer have prepared platforms (Table 3). Retouched artefacts constitute 1.2% of the flake assemblage (Table 3) and include notches, denticulates, burins and scrapers. Raw material distributions are similar to the Bedded Sands member with quartzite dominant, followed by chert and then non-local chert (Table 4). A refit between two artefacts from these two members accords with their similarity in technology and raw material distribution and the observation that bioturbation has mixed the boundary of these deposits [37]. This suggests that in accordance with Waechter [9], the artefacts from these upper Bedded Sands and the lower Upper Bioturbated Sands should be viewed as a single assemblage.

Towards the back of Gorham’s Cave new excavations have uncovered Mousterian artefacts in a young deposit known as Layer IV [12]. Layer IV has been dated via radiocarbon to between 35 and 28 kya [5], [6]. The cores from Layer IV include four single platform cores, one discoidal core, one facetted centripetal disc, and two broken cores. The occasional presence of flakes with facetted platforms (Table 3) and radial dorsal scar patterns accords with the presence of a facetted centripetal disc core. Retouched pieces include scrapers and denticulates and comprise 6.7% of the flake assemblage (Table 3). No non-local cherts are present in the raw materials with this assemblage more dominated by coarser grained quartzites than any other Mousterian layer from Gorham’s Cave (Table 4).

### Comparative Analyses

Here we statistically assess the lithic patterns described above, including the variation in reduction techniques and raw material exploitation across the Mousterian of the Gibraltar Caves. We examine the diachronic variation in raw material selection, flake platform preparation and core reduction technology through the Gorham’s sequence.

**Figure 3:**
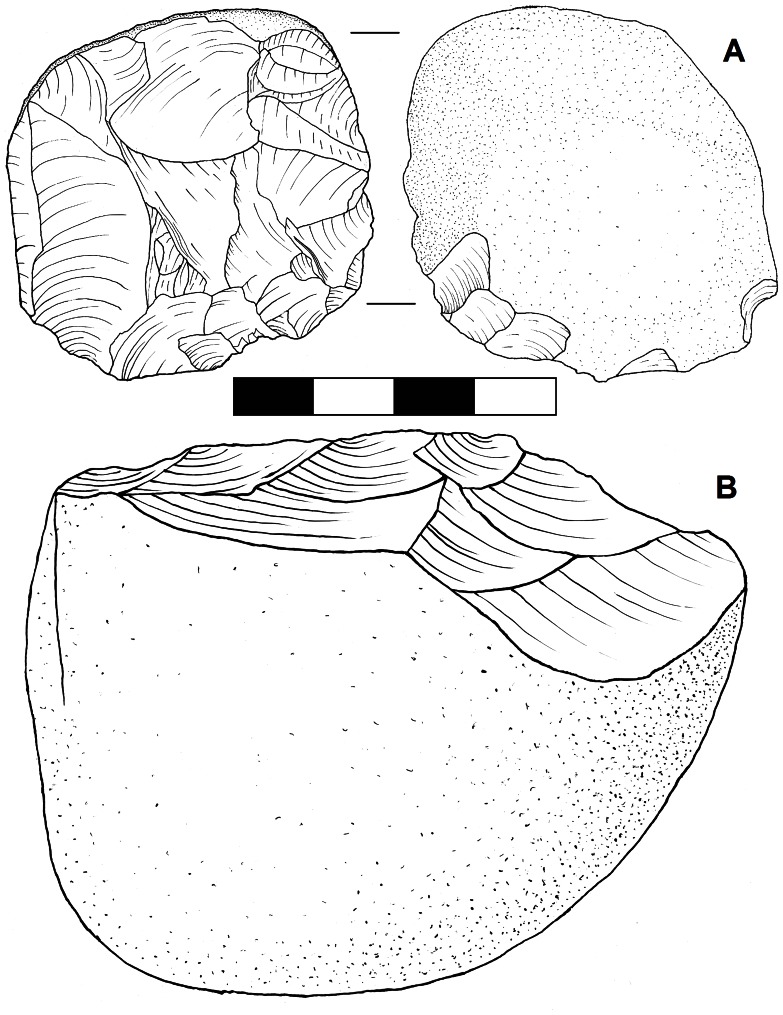
Two Mousterian cores from Gibraltar. A: facetted centripetal disc core on rounded dark red chert from Gorham’s Cave Lower Bioturbated Sands member GOR95 200; B: quartzite unifacial cobble core from Gorham’s Cave Layer IV GOR00 72.

**Figure 4:**
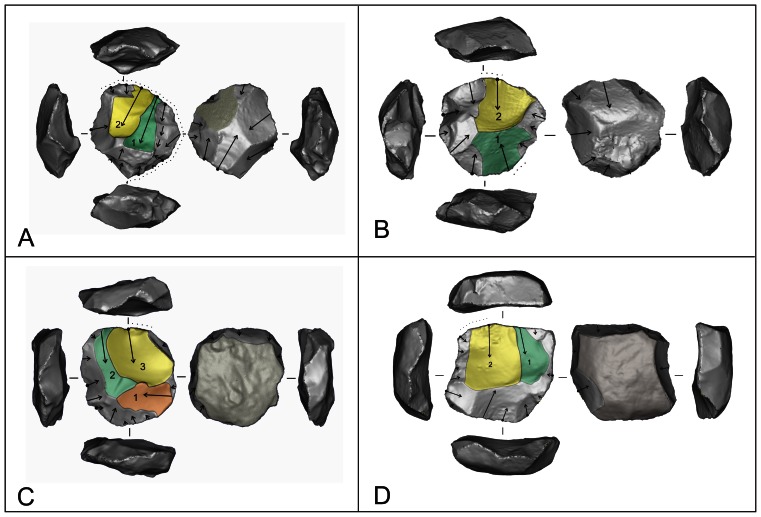
Levallois cores from Gibraltar. A: Gorham’s Cave Waechter’s Layer P, recurrent unidirectional Levallois on chert. B: Gorham’s Cave Sands and Stony Lenses member GOR98 925, recurrent bidirectional Levallois on chert. C: Gorham’s Cave Waechter Layer M, recurrent centripetal Levallois on chert. D: Ibex Cave 046, recurrent unidirectional Levallois on jasper.

A total of 54 cores and assayed clasts from the four Gibraltar caves described above (consisting of all cores available at the Gibraltar Museum at the time of data collection) were examined to quantify patterns in stone reduction technology in the Gibraltar Mousterian. The Middle Palaeolithic cores of Gibraltar are typologically characteristic of Neanderthal technology elsewhere e.g. [38], [21], ranging from more formal Levallois and discoidal cores to less formal multi-platform and single platform cores (Figure 3). The Levallois cores cover a range of sub-types including preferential centripetal, recurrent unidirectional, recurrent bidirectional and recurrent centripetal (Figure 4). The informal single and multi-platform cores have lower flake scar densities, greater masses, higher platform angles, and larger platforms than the formal Levallois and discoidal cores (Table 5). Mann-Whitney U tests showed these differences were significant at the P = 0.005 level. Informal cores are thus flaked expediently without maintaining low platform angles and exploiting small platforms for long term reduction, so they are discarded while they are still large with fewer flake removals. The informal cores tend to be made on the coarser grained quartzite (56%) and limestone (11%), while the formal cores tend to be made on varieties of chert (77%) (Table 6). A chi-squared test showed this difference to be significant at the P = 0.05 level, indicating greater investment was put into the use of the more spatially restricted and higher quality raw material.

**Table 5:** Mann-Whitney U tests comparing flake scar density in scars per cm^2,^ weight in grams, platform angle in degrees and platform size in mm^2^ between formal and informal cores.

	Formal	Informal	Significance
**N**	27	10	
**Median Flake Scar Density**	0.6	0.3	
**Mean Scar Density Rank**	22.07	10.7	0.004
**Median Mass**	28	86	
**Mean Mass Rank**	15.91	27.35	0.003
**Mean Platform Angle**	74	90	
**Mean Angle Rank**	16.04	27	0.005
**Mean Platform Size mm^2^**	531	1771	
**Mean Platform Size Rank**	16.52	25.7	0.021

**Table 6:** Breakdown of raw material type for formal and informal cores from Gibraltar.

	Formal	Informal	Total
**Limestone**	1 (3.2%)	2 (11.1%)	3
**Quartzite**	5 (19.3%)	10 (55.5%)	15
**Chert**	24 (77.4%)	5 (33.4%)	29
**Total**	30 (100%)	17 (100%)	47

For the Chi-square test limestone and quartzite cores were lumped together and Yates’ continuity correction was applied.

The largest flakes from Gibraltar are made on the non-local honey coloured chert. To assess the differences in the use of this chert in comparison to the local varieties available on The Rock we examined artefact type frequencies from Gorham’s Cave (Table 7). A chi-squared test showed these differences to be significant at the P<0.0001 level (retouched flakes were grouped with other flakes for this analysis due to the small sample size), indicating that there are proportionally more Levallois and radial flakes, and core edge removal flakes in the honey chert, and proportionally less cores. This indicates that Levallois products were preferentially manufactured and/or transported on the honey chert, while the honey chert cores were likely more formal and rejuvenated so they had longer use lives than the local chert. Interestingly, the level of retouch does not appear to be higher for the honey chert. There also appears to be no association between the levels of retouch and the use of informal or formal core technology across the Gibraltar assemblages. Given the abundance of raw materials on Gibraltar, frequent retouching to resharpen edges was probably unnecessary, and retouching was instead employed to create specific edge shapes such as concave notches and denticulates, and steep scraper edges.

**Table 7:** Breakdown of different flake types for the non-lcoal honey coloured chert and local chert from Gorham’s Cave.

	Honey Chert	Other Chert
**Levallois and Radial Flakes**	17 (31%)	31 (8%)
**Core Edge Removal Flakes**	7 (13%)	22 (6%)
**Retouched Flakes**	3 (5%)	20 (5%)
**Other Flakes**	27 (49%)	283 (74%)
**Cores**	1 (2%)	27 (7%)
**Total**	55 (100%)	383 (100%)

Data from Barton & Jennings [14].

In Gorham’s Cave a pattern was observed whereby the use of more local and coarser grained materials appears to increase through time. Limestone is available in the cave itself, while quartzite occurs both as beach cobbles and as a now submerged primary outcrop in front of the cave. Chert, whilst sometimes procured as small beach pebbles, is generally more spatially restricted on The Rock, with some chert even procured from inland. We use a series of Fisher’s Exact tests to compare the proportions of coarser grained limestone and quartzite, with finer grained chert (Table 4), between each successive stratigraphic layer. Between the Sands and Stony Lenses member and the overlying Lower Bioturbated Sands member the decrease in chert was significant (P = 0.0062). Between the Lower Bioturbated Sands member and the overlying Bedded Sands member the decrease in chert is significant at the P<0.0001 level. Between the Bedded Sands member and the Upper Bioturbated Sands member there is no significant difference in the proportion of chert and coarser materials (P = 0.7155). Between the combined Bedded Sands and Upper Bioturbated Sands member, and Layer IV there is a borderline difference in the decrease in chert (P = 0.0853), although it is noteworthy that there is no non-local chert in Layer IV.

Platform preparation is a parameter of investment in flake production, with higher proportions of platform preparation reflecting more formal production of flakes. Using Fisher’s Exact tests we assess the pattern of decreasing platform preparation through time in Gorham’s Cave, by comparing the proportion of platform preparation in sequential levels (Table 8). There is a significantly higher degree of platform preparation in the Sands and Stony Lenses member (including Waechter’s layers L, M, O and P) when compared with the overlying Lower Bioturbated Sands member (including Waechter’s layer K) (P<0.0001). There is no significant difference in the degree of platform preparation between the Lower Bioturbated Sands (including Waechter’s layer K) and the Bedded Sands member (P = 0.7832). The proportion of faceting in the Upper Bioturbated Sands member (including Waechter’s layer G) is significantly lower than the Bedded Sands member (P = 0.0001). There is no significant difference in the proportion of faceting between the Upper Bioturbated Sands member and Layer IV (P = 0.1108). There are then two significant drops in the proportion of platform preparation moving upwards through the Gorham’s Cave sequence. This pattern is also reflected in the cores with a Fisher’s Exact test showing significantly fewer instances of platform preparation in the cores from Layer IV and the Upper Bioturbated Sands member in comparison to those from the Lower Bioturbated Sands member and the Sands and Stony Lenses member (N = 24, P = 0.0381). The only core to exhibit both overhang removal and platform faceting from Gorham’s Cave is also from the Sands and Stony Lenses member.

**Table 8:** The frequencies of unprepared and prepared platforms on flakes from Gorham’s Cave.

	Prepared	Unprepared	Total
**Layer IV**	3 (9%)	29 (91%)	32 (100%)
**UBSm**	202 (4%)	5006 (96%)	5208 (100%)
**BeSm**	15 (12%)	106 (88%)	121 (100%)
**LBSm**	196 (14%)	1216 (86%)	1412 (100%)
**SSLm**	434 (21%)	1619 (79%)	2053 (100%)

Data for UBSm, BeSm, LBSm and SSLm from Waechter [9] and Barton & Jennings [14].

It has been suggested that the occupation of the Layer IV of Gorham’s Cave represents the early Upper Palaeolithic [35], in which case we would expect a significant shift towards blade technology. The core typology belies this hypothesis as there are no blade cores from the upper area of the cave, while there is continuity between Layer IV and the middle area in the presence of facetted disc, discoidal and single platform cores. In fact not a single blade scar was observed on any of the cores from the upper area of Gorham’s Cave. Correspondingly Pacheco *et al.* [12] found that the Layer IV flakes had a blade index of 1.2 indicating blades are not typical for this assemblage.

To obtain a statistical overview of the technological variation in the Gibraltar Mousterian we measured a suite of variables on the lithic cores. To put the Gibraltar cores in context we also measured cores from two classic Middle Palaeolithic sites: Le Moustier in France, the type site of the Mousterian, and Tabun Layer C, one of the best known Levantine Middle Palaeolithic assemblages. The variables measured were as follows: the percent of cortex remaining on the core; the number of flake scars; the proportion of blade scars; the length to width ratio of the core (oriented along the main axis of flaking); the width to thickness ratio of the core; the ratio of the proximal width to the distal width of the core; the lateral and distal curvature of the upper surface; the relative intersection height of the main flaking surface and the underlying surface; the mean platform angle; the number of platforms; the proportion of the perimeter of the upper core face that was faceted; the proportion of the core face covered by the length of the largest scar; and the scar pattern angle of the upper and lower faces [39], [40], [41]. A Principal Components Analysis was then conducted on these variables to tease out any underlying patterns. Broken cores and assayed clasts (cores with less than 4 deliberately initiated flake scars) were excluded from the analysis to avoid missing data. The total sample entered into the analysis was 100 cores. Some variables were transformed to give them a normal distribution in accordance with the assumptions of the analysis, namely: the proportion of faceting; the number of scars; the proportion of blade scars; length to width ratio, width to thickness ratio, the scar pattern angles; the percentage of cortex and the number of platforms. Bartlett’s Test of Sphericity was significant at the P<0.001 level, indicating correlations between individual variables, and the Kaiser-Meyer-Olkin measure of sampling adequacy was 0.702, indicating there are correlations between pairs of variables and other variables, therefore a factor analysis is appropriate.

**Figure 5:**
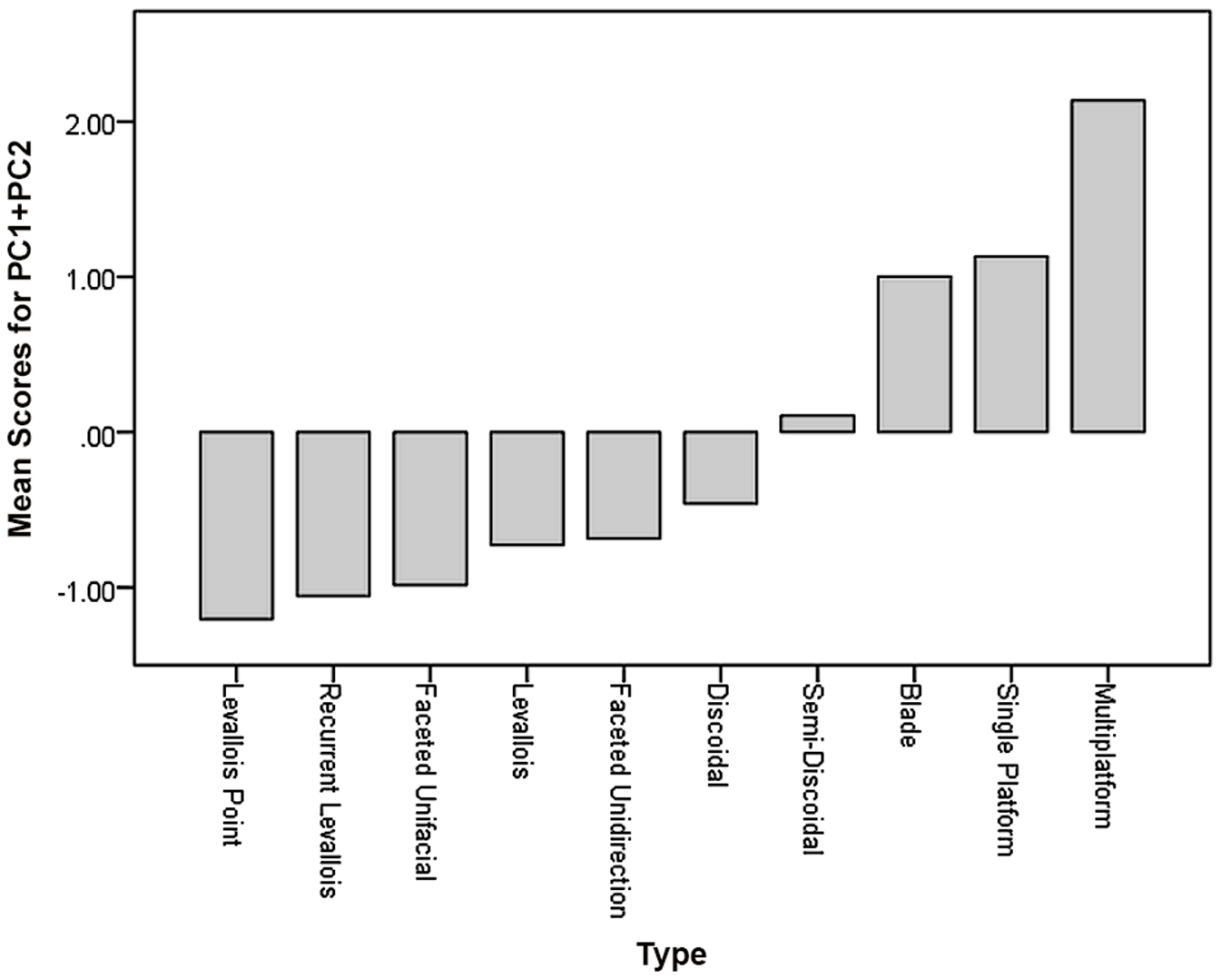
Mean summed component 1 and 2 values for different core types. Note that unidirectional and multiplatform technologies tend to have positive values, while radial technologies tend to have negative values.

**Figure 6:**
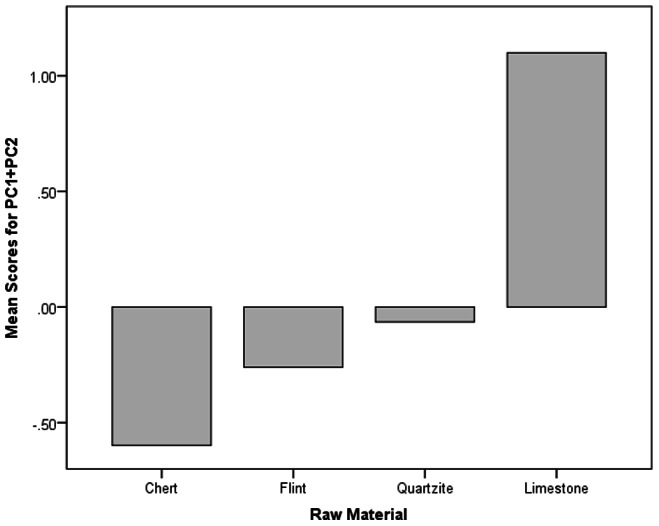
Mean summed component 1 and 2 values for different raw materials. Note that coarser grained materials tend to have higher values and finer grained materials tend to lower negative values.

Four components were extracted with Eigenvalues over 1, hence these factors explain a greater proportion of the variance in the input variables than any individual input variable. The first two components accounted for 28.9% and 18.5% of the variance respectively, so almost half the variance in the input variables is explained by these two components. The component matrix (Table 9) shows that higher values of component 1 denote cores which have flat upper surfaces and a high point of intersection between the main surface and the surface below; they are relatively narrow and thick; they have high proportions of blade scars and high platform angles; the largest scar runs across a high proportion of the core face; the scar patterns on the main flaking surface are parallel; and they have few scars. Component 1 thus distinguishes between unidirectional reduction methods and bifacial radial technologies. Cores which have high values of component 2 tend to have a high number of separate platforms; high numbers of scars, multi-directionally flaked upper and lower surfaces; low cortex coverage; low proportions of blade scars; low proportions of faceting; high platform angles; and short largest scars. Component 2 thus distinguishes between multi-platform cores and more systematic Middle Palaeolithic technologies. Figure 5 shows that core types based on unidirectional or multiplatform flaking have positive values for the summed components 1 and 2, while technologies based on radial flaking have low values for components 1 and 2. Figure 6 shows variation in components 1 and 2 by raw material and indicates that the coarser grained limestone is associated with more expedient unidirectional and multiplatform flaking, while the finer grained flints and cherts and associated with systematic radial flaking, and quartzite is intermediate between the two.

**Table 9:** The Matrix of the first two components in the Principal Component’s Analysis.

Input Variable	Component 1	Component 2
**Percent of Cortex**	0.065	−0.552
**Number of Scars**	−0.29	0.617
**Proportion of Blade Scars**	0.688	−0.459
**Length to Width Ratio**	0.736	0.219
**Width to Thickness Ratio**	0.808	0.14
**Proximal to Distal Width Ratio**	0.003	−0.22
**Upper Surface Curvature**	−0.9	−0.177
**Relative Intersection Height**	−0.873	−0.161
**Mean Platform Angle**	0.471	0.41
**Number of Platforms**	0.181	0.666
**Proportion of Faceting**	−0.059	−0.438
**Largest Scar Proportion**	0.517	−0.245
**Upper Scar Pattern Angle**	−0.45	0.501
**Lower Scar Pattern Angle**	−0.048	0.636

**Figure 7:**
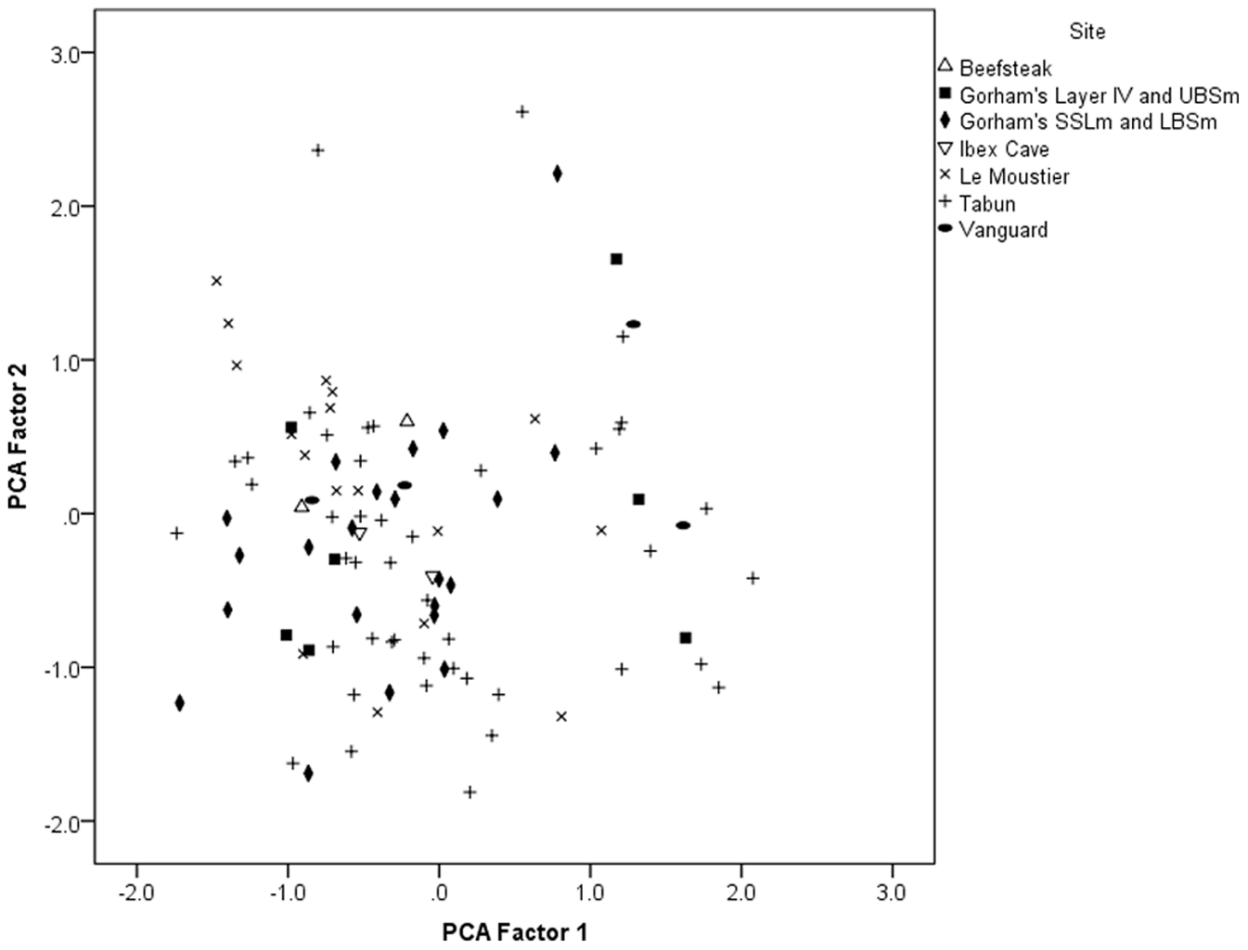
Scatter plot of the first two principal components. Note that most cores are concentrated in the bottom left of the distribution. Both the two cores from Beefsteak Cave and the two cores from Ibex Cave occur close to each other. The cores from Vanguard Cave and the cores from Gorham’s Layer IV and UBSm are not concentrated in the bottom left of the distribution, indicating their more expedient nature.

A scatter plot of the first two principal components shows how the cores from each assemblage are distributed (Figure 7). Most of the cores in the analysis fall into the bottom left of the distribution, reflecting the systematic radial nature of Levallois and discoidal based Middle Palaeolithic technology. The collection strategies at Le Moustier and Tabun C may have been somewhat biased towards more formal core types. There are however, significant numbers of cores that are more unidirectional on the right of the graph, and multi-platform on the upper part of Figure 7. The small sample of cores from Vanguard are widely spread but they tend to occur towards the top right of the distribution reflecting their relative emphasis on expedient flaking. The two cores from Beefsteak Cave occur near each other as to do the two cores from Ibex Cave reflecting the similarity in technology within each cave. The majority of cores from Gorham’s SSLm and LBSm are clustered in the bottom left, reflecting the dominance of Levallois and discoidal flaking in the early phases of occupation in the cave. The cores from Gorham’s Layer IV and Gorham’s UBSm are similarly distributed to those from Vanguard, being widely dispersed and not clustered in the bottom left of the distribution; therefore they tend to be more expedient than the cores from the lower members of Gorham’s Cave.

## Discussion

The Mousterian technology of Gibraltar documents the use of the caves by Neanderthal populations during MIS 3, 4 and 5. The homogeneity in technology in Beefsteak and Ibex Caves and the presence of refits in the small assemblage from the latter, suggests that the occupation of these caves may be ascribed to single episodes. On the other hand, Vanguard Cave contains a longer sequence of stratified occupations with a greater variety of lithic assemblages; yet, low artefact densities and the presence of refits illustrates that individual occupations were relatively short-term. This accords with the evidence that the hearths at Vanguard were either used once, or used, abandoned and later reused [42]. Gorham’s Cave has a denser, more continuous sequence of lithic artefacts, with greater organic content in its sediments, corroborating the notion that the cave was more heavily utilized [42], [43]. The paucity of large vertebrate remains from Gorham’s Cave has been interpreted to be a consequence of housekeeping, thus implying longer-term occupation [44]. Gorham’s Cave is the largest of the four caves, and its high ceiling and exposure to sunlight make it the most suitable for habitation [5]. The presence of numerous fine sub-strata in Gorham’s Cave and the spatially clustered distributions of lithics with refits, suggests that this sequence is composed of many short phases of occupation. The dichotomy in occupation intensity between Gorham’s and the other caves, suggests the southern Iberian Neanderthals may have practised a mobility pattern in which hominins would temporarily occupy various sites during the course of foraging, but would regularly return to a particular hub locality, such as Gorham’s. A similar pattern of radiating mobility has been suggested for the Levantine Neanderthals [45], [46], [47].

In general we may describe three technological strategies employed by the Neanderthals of Gibraltar. The most formal involves Levallois reduction of large clasts of honey coloured chert from inland Iberia. Large Levallois flakes and some Levallois cores in this honey chert were then selected and carried over a distance of at least 17km to Gibraltar. The intermediate strategy comprises the exploitation of chert and quartzite from outcrops on and around The Rock by Levallois and discoidal reduction techniques, often with platform preparation. The third strategy involves the expedient single and multiplatform reduction of quartzite cobbles and chert pebbles from the beaches in front of the caves, or even using the limestone of the caves themselves. All three strategies are evident in the earliest dated occupation phases on Gibraltar from Vanguard Cave. The ephemeral Beefsteak and Ibex Cave occupations are characterised by the intermediate strategy. In Gorham’s Cave there appears to be a diachronic trend with the earlier levels focussed on the more formal strategies; then a shift towards expedient strategies in the later levels, with no non-local honey coloured chert unknown in the final phase of Mousterian occupation.

The formal cores are significantly smaller and have higher flake scar densities than the informal cores, indicating they were more heavily worked. The three strategies appear to reflect different mobility patterns as the most formal technology is practised on the non-local material and the most expedient technology is used on the most immediately available material. Several researchers have correlated expedient forager technology with low mobility and formal forager technology with high mobility e.g. [16], [21], [48], [49], [50], [51]. Expedient technologies opportunistically create flakes from locally available or stockpiled stone, without the emphasis of production on standardized and functionally generalized tools. Typically, little effort is dedicated to core preparation and little prior technical knowledge is required. Since expedient core reduction techniques are less complex they can be easily adapted to a variety of raw materials of varying quality that may be immediately available. In the Levantine Middle Palaeolithic, assemblages with more expedient technology are associated with long-term, relatively sedentary Neanderthal occupation [21]. Formal technologies on the other hand aim at maximizing the size and standardizing the shape of flake blanks and with the greater investment in technology usually correlated with selection of high quality materials [17], [50], [51], [52], [53], [54]. Formalising reduction sequences to manufacture standardized blanks may be desirable when tools are manufactured for future use at times of limited opportunity for resupply, such as during periods of high mobility. In south-west France for example Mousterian artefacts from the most distant raw material sources are mostly formal Levallois products [55].

A GIS analysis of the Southern Iberian Mousterian showed that sites are concentrated both near the coast and along major rivers [56], [57]. The climate of the coast and major river valleys tends to be warmest, wettest and most stable, resulting in a diversity of habitats [56]. During MIS5 these favourable habitats would have been expansive, and it is from this time that we have the earliest evidence for occupation on Gibraltar.

The optimal area for Late Pleistocene hominin occupation in southern Iberia, with the highest rainfall and temperature, and the greatest stability and diversity, would have been Gibraltar and its immediate environs [56], [57], [58]. The herpetofauna from Gibraltar shows no evidence for extended cold conditions during MIS5-3 [59], [60], while the small and large mammal fauna are also remarkably stable and representative of inter-glacial conditions [44], [61], [62]. Gibraltar may thus be described as a refugium with its stable and diverse habitat [6], [63], [64]. The diverse habitat of Gibraltar is reflected in the wide variety of food resources exploited by the Gibraltarian Neanderthals; including red deer, ibex, wild boar, rabbits, seals, dolphins, birds, tortoises, fish, shellfish and pine nuts [3], [4], [65], [66].

Bio-climatic modelling indicates that the favoured habitats of the southern Iberian Neanderthals became fragmented during MIS3 separating coastal and upland populations [56], with much of the interior of Iberia becoming arid [67]. The MIS3 occupation of Gorham’s Cave may represent a Neanderthal population which foraged locally along the coast and did not exploit inland resources to the same extent as their predecessors had done. Indeed, the low seas level stands of MIS3 would have opened up new shore habitats immediately in front of Gibraltar [58], [59]. This may explain why the technology becomes increasingly expedient and made on more local materials during the later occupation phases at Gorham’s, reflecting reduced residential mobility and greater emphasis on foraging on and around The Rock. Waechter’s layer G has a far higher artefact density than the underlying layers, suggesting more intensive occupation, perhaps as a result of a reduced residential mobility pattern [68]. An increase in the quantity of charred material in the MIS3 occupation of Gorham’s also indicates more intensive hominin occupation at this time [69].

Parallels may be found with MIS3 Neanderthals populations elsewhere. In the southern Caucasus the environment was stable and diverse, like Gibraltar, and also did not suffer the MIS3 deterioration to the same extent as surrounding regions [70]. In the late Middle Palaeolithic of the southern Caucasus, prior to replacement by *Homo sapiens* c. 37kya, there was also a reduction in Neanderthal range size with far fewer exotic materials being exploited than in the earlier Middle Palaeolithic [20]. In the Middle Palaeolithic of Latium, Italy, the onset of MIS3 coincided with a reduction in the import of exotic materials and a shift away from radially prepared cores for striking larger flakes, to bidirectional small flake cores [19].

Gibraltar has been hypothesized to be one of the last refuges of the Neanderthals with a date of 28 kya for the youngest Mousterian occupation in Layer IV of Gorham’s Cave [5] [6]. This young age has been challenged partly on technological grounds with the suggestion that the Layer IV occupation of Gorham’s actually represents the early Upper Palaeolithic rather than the latest Mousterian [35]. However, there are no blade cores, or even blade scars on the cores from Layer IV. There is also continuity between Layer IV and the older members of Gorham’s Cave in the use of large rounded quartzite cobbles as single platform cores and in the production of small discoidal cores on chert. In accordance with previous analyses [12] we must therefore assign the Layer IV artefacts to the Mousterian. The absence of any Levallois cores from this final occupation could reflect a dwindling population in which the expertise required for this most complex of Middle Palaeolithic technologies has been lost (*sensu* Henrich, [71]), but larger samples are needed to test this hypothesis.

The Mousterian record from the Gibraltar caves provides a rich sequence of Neanderthal occupation in an optimal habitat. The high biodiversity and stability of the Gibraltar climate may have allowed this region to act as a refugium for the last surviving Neanderthals [63], [72], [73]. As the climate deteriorated during MIS3 the technological response of the Neanderthals was to use more expedient flaking strategies on locally available material, reflecting a reduction in mobility and a contraction into the core zone of the refugium.
